# American Dentists Not Wanted in Germany

**Published:** 1897-03

**Authors:** 


### American Dentists not Wanted in Germany.

A dentist was recently arrested and fined, in Berlin, for displaying upon the door of his office a plate describing himself as a doctor of dentistry, with a diploma granted by an American dental college. The court held that it was against the law for him to use a foreign title in practice in Germany.

What a free and happy country is that known as the British Isles. Everywhere these American dentists abound, with their dental diplomas, by which they are able to prevail upon the British public to patronize them. America is, undoubtedly, a great country for manufactured articles of a kind, but so far, at all events as dentist are concerned, the latter must be " made in Germany," if they desire to practice their profession in the Fatherland. As, however, Englishmen have only a " John Bull" to look to and not a " Fatherland," and a great deal is required to arouse the old gentleman to the responsibility of his position, an alien in this country has, usually, a good time of it all round.—*Med. Press and Circular.*

